# The Pilonidal Sinus Disease-Specific Quality of Life Questionnaire (SQoL): Reliability, Validity, and Utilisation

**DOI:** 10.3390/jcm15155875

**Published:** 2026-07-27

**Authors:** Edvinas Dainius, Monika Vaiciute, Edgaras Burzinskis, Audrius Parseliunas, Tadas Latkauskas, Violeta Simatoniene, Silvija Ilgunaityte, Donatas Venskutonis, Algimantas Tamelis

**Affiliations:** 1 Department of Surgery, Lithuanian University of Health Sciences, LT-44307 Kaunas, Lithuania; edvinas.dainius@lsmu.lt (E.D.); monika.vaiciute@kaunoklinikos.lt (M.V.); edgaras.burzinskis@lsmu.lt (E.B.); tadas.latkauskas@lsmu.lt (T.L.); donatas.venskutonis@lsmu.lt (D.V.); 2 Hospital of the Lithuanian University of Health Sciences, LT-50161 Kaunas, Lithuania; audrius.parseliunas@gmail.com; 3 Independent Researcher, LT-44307 Kaunas, Lithuania; violsim@gmail.com; 4 Lithuanian University of Health Sciences, LT-44307 Kaunas, Lithuania; silvija.ilgunaityte@stud.lsmu.lt

**Keywords:** pilonidal sinus disease, quality of life, quality of life questionnaire, disease-specific quality of life questionnaire

## Abstract

**Background:** Pilonidal sinus disease is an inflammatory condition characterised by formations ranging from minor cysts to extensive sinus tracts in the natal cleft in the sacrococcygeal area. Due to its complex and unique symptoms, the condition may not be adequately captured by the generic quality of life (QoL) instruments. In this study, we evaluated a newly developed pilonidal sinus disease-specific quality of life (SQoL) questionnaire, valuating its validity and applicability for measuring postoperative outcomes of pilonidal sinus disease. **Materials and Methods:** The study involved developing a SQoL. A total of 116 adult patients with chronic and acute symptomatic disease at the Kaunas Hospital of the Lithuanian University of Health Sciences (LUHS) were asked to complete both the SQoL and the SF-36v2 questionnaire. Key evaluation criteria for the SQoL questionnaire included internal consistency, measurement stability, as well as construct and discriminant validity. **Results:** 2 months after the procedure, 103 patients (response rate 88.79%) completed both questionnaires. Internal consistency was assessed 1 week after surgery, with the overall questionnaire achieving a Cronbach’s α coefficient of 0.919. The Spearman correlation coefficient for the total questionnaire score in the test–retest validation was 0.55 (CI 0.406–0.679), indicating a moderately significant correlation for all questions. Construct validity was evaluated by comparing the SQoL questionnaire with the results from various SF-36v2 domains 1 week after surgery. The highest inverse correlations were found between the corresponding domains of Physical Functioning and Role Physical Functioning (−0.770 and −0.600), with all correlations being statistically significant. **Conclusions:** A newly developed SQoL questionnaire has shown promise in assessing the impact of the disease and its treatment, particularly by addressing disease-specific symptoms. Standardising outcome measures can improve the reliability of meta-analyses and systematic reviews, which in turn helps to create more personalised care plans. The trial was registered in ClinicalTrials.gov with the identifier of NCT05982028.

## 1. Introduction

Pilonidal Sinus disease (PSD) is an inflammatory condition that can manifest as either acute or chronic, ranging from minor cysts to extensive sinus formations [1]. This condition affects the natal cleft in the sacrococcygeal area, with an incidence of 26–48 cases per 100,000 population, and is 2.2 times more prevalent in young males than females [2,3]. Chronic pilonidal abscesses are characterised by pain, fistula development in the sacral region and secretion. These symptoms can significantly disrupt daily activities, negatively impact sexual function, and lead to absences from work, thereby reducing quality of life [4,5].

The main treatment for chronic pilonidal sinus disease is surgery, with different methods or modifications of these methods being used worldwide [6,7]. It is suggested that the ideal surgery should be simple and brief, with a short hospital stay and recovery period, a swift return to normal activities, low rates of recurrence and unsuccessful outcomes, and a favourable cosmetic result [8]. However, any of these surgical procedures can affect patients’ psychological and physical well-being and significantly impact self-reported quality of life [9,10].

Quality of life (QoL) outcomes are the most commonly used evaluation method for determining treatment efficacy [11]. One commonly utilised tool for evaluating the impact of diseases on patients’ QoL across diverse patient groups and conditions is the SF-36v2 (Short Form-36 Health Survey version 2) questionnaire. It measures each of the following eight health domains: physical functioning, role-physical, bodily pain, general health, vitality, social functioning, role emotional, and mental health [12]. Due to its thoroughness and capacity to deliver reliable and valid QoL measurements, the SF-36v2 is widely employed in clinical studies and health outcomes research [13].

Assessing QoL after pilonidal sinus disease surgery is crucial for understanding the holistic impact of the disease and the treatment on patients. Currently, various generic QoL assessment tools like visual analogue scale (VAS) and SF-36v2 are widely used in medical research to measure patients’ well-being across different health conditions. However, the unique challenges and symptoms associated with PSD may not be adequately captured by these generic instruments, as highlighted in recent articles [14,15].

The aim of this study was to develop a pilonidal sinus disease-specific QoL (SQoL) questionnaire, evaluate its validity and suitability compared with the standard SF-36v2 outcomes.

## 2. Materials and Methods

### 2.1. Study Protocol and Patient Selection

The developed QoL questionnaire was used at the Kaunas Hospital in a single-centre trial that took place from 1 September 2020 to 1 February 2023, when the necessary number of participants was reached. The study protocol was approved by the local Medical Ethics Committee in accordance with the principles of the Declaration of Helsinki and is available at LUHS’s Department of Surgery.

The sample size was based on commonly accepted recommendations for patient-reported outcome measure (PROM) validation studies, which suggest including at least five participants per questionnaire item. Since the SQoL questionnaire contains 20 items, a minimum of 100 participants was required. To allow for potential patient loss and incomplete responses, we increased the planned recruitment target by 20%, resulting in an intended sample size of 120 patients.

The patient inclusion criteria were as follows: (1) age between 18 and 75 years, (2) chronic and acute symptomatic pilonidal sinus disease (primary or recurrent), (3) physical status I to III according to the American Society of Anesthesiologists (ASA), and (4) signed informed consent to participate in the study.

Patients were deemed ineligible if they met any of the following criteria: (1) had asymptomatic sinus, (2) non-Lithuanian speakers, (3) had cognitive, visual, auditory, or locomotor system disorders, (4) had significant kidney, liver, or cardiopulmonary insufficiency, or (5) refused to participate in the study.

Patients who met the inclusion criteria underwent one of the PSD surgeries: abscess incision and drainage, minimally invasive pit-picking surgery, radical sinus excision without suturing, or excision according to Karydakis. They were asked to fill out the SQoL questionnaire and SF-36v2 at the following time points: before surgery, during the first and second weeks post-surgery, and two months after surgery. Patients were scheduled for a follow-up consultation at two and six months post-surgery to assess wound healing. At the six-month follow-up, patients completed the SQoL questionnaire to evaluate its potential as an indicator of unsuccessful surgical outcomes.

The primary objective of this study was to assess the validity and suitability of a newly developed pilonidal sinus disease-specific quality of life questionnaire. The secondary objective was to assess quality of life using the SQoL questionnaire and the SF-36v2 questionnaire.

### 2.2. Assessment

#### 2.2.1. Disease-Specific Quality of Life Questionnaire (SQoL)

The entire process involved three stages: draft, modification, and confirmation.

##### Draft Stage

A SQoL was developed based on previous publications and clinical experience related to the symptoms experienced by patients after PSD surgeries.

The questionnaire was designed to evaluate four key areas that have the greatest impact on quality of life: pain, physical activity, symptoms related to the wound, and psychosocial well-being. Each SQoL subscale consists of 5 questions.

The pain subscale includes questions about pain at rest, while walking, during daily activities, and during moderate-intensity activities. It also assesses pain during specific movements that may exacerbate discomfort in the sacral region, such as sitting on the toilet. Patients were required to rate the intensity of pain for each question using a scale ranging from no pain to very severe, corresponding to scores from 0 to 5, respectively.

The physical activity subscale includes questions related to changes in physical activity levels during walking, stair climbing, and participation in low, moderate, and high-intensity activities.

The wound-related symptoms subscale includes questions about wound secretion, odour, dressing management, limitations in movement due to the wound or dressing, and the requirement for additional personal assistance in wound care.

In the final psychosocial subscale, patients are asked questions about their emotional well-being, engagement in work and leisure activities, and avoidance of close relationships. Specific questions about changes in sexual activity were also included.

The questions in the wound-related symptoms, physical activity and psychosocial state subscales were assessed using a Likert scale ranging from 0 to 4. Higher scores indicate poorer QoL. The questionnaire has a minimum total score of 0 points, indicating very good QoL, and a maximum score of 85 points, indicating very poor QoL.

##### Modification Stage

In the second phase, the questionnaire was reviewed by two expert coloproctologists and a general surgeon to assess its suitability. Following the evaluation by medical experts, several statement formulations were revised, while the overall structure of the questionnaire remained unchanged. Furthermore, the questionnaire was administered to 10 patients with PD to ensure that its language was appropriate and understandable.

##### Confirmation Stage

**Figure 1:**
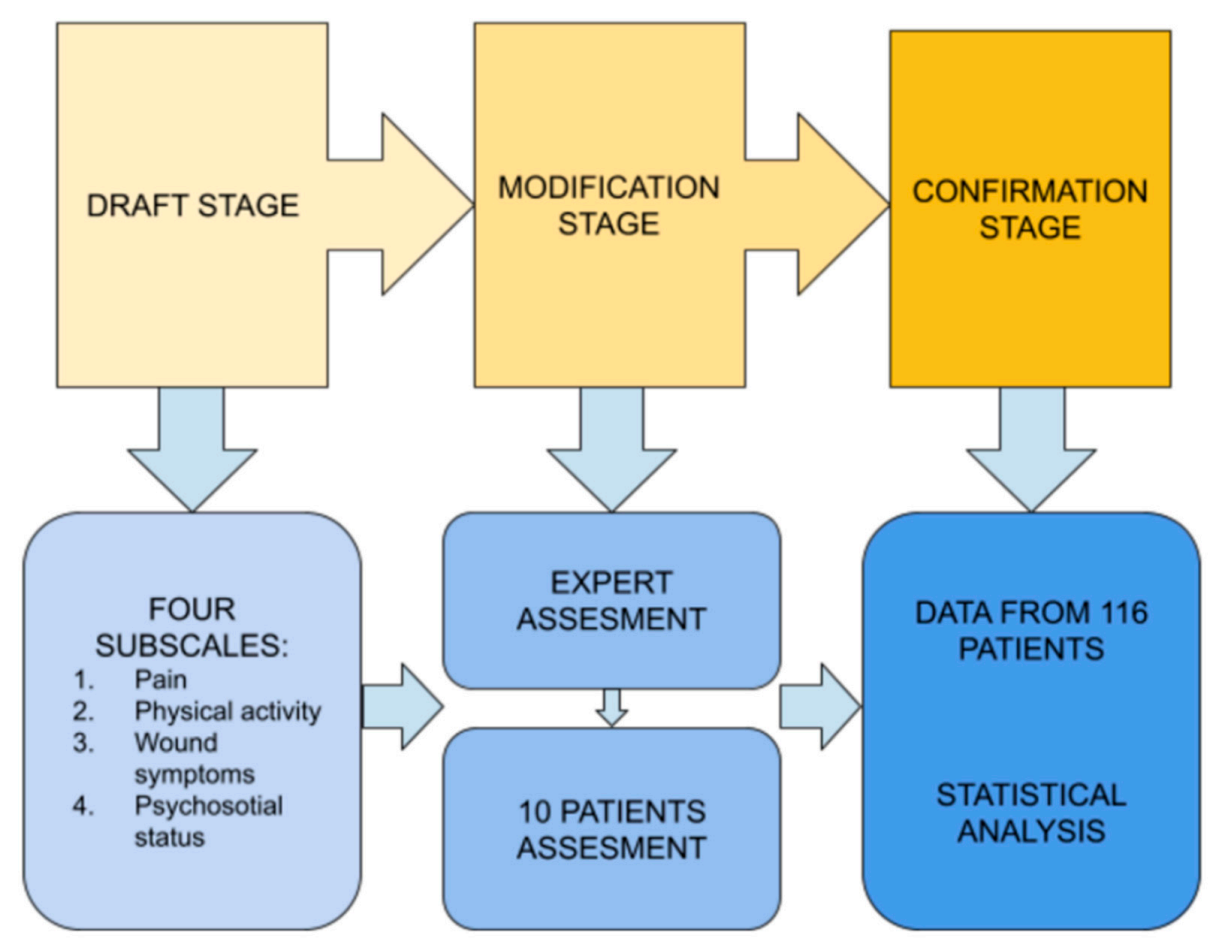
SQoL development process.

At this stage, we surveyed 116 patients after PSD surgery and conducted a statistical analysis. To ensure that each question demonstrated independence, high sensitivity, and representativeness, we performed an analysis of all 20 questions to identify and exclude any unsuitable ones. Since no such questions were identified in the questionnaire, we proceeded with construct validity and reliability analyses. The entire SQoL development process is illustrated in Figure 1.

The final version of the SQoL questionnaire is provided in the Supplementary Materials (Supplementary Table S1).

#### 2.2.2. Statistical Analysis

The statistical analysis was performed using SPSS for Windows, version 29 (IBM Corp, Armonk, NY, USA). A significance level of *p* < 0.05 was used. Baseline characteristics and demographic data were analysed descriptively. Continuous data are presented as mean and standard deviation, while categorical data are presented as a percentage. The Shapiro–Wilk normality test was performed for continuous variables. Since all analysed data were found to be non-normally distributed, the Mann–Whitney U test was utilised to compare continuous variables with non-normal distributions. Quantitative variables are presented as medians (Q1–Q3).

Acceptability was measured as the proportion of returned and fully completed questionnaires. Questionnaires with at least one missing item were considered incomplete.

Internal consistency was assessed using the Cronbach α coefficient, which summaries the intercorrelations of all items in a scale and each of the 4 subscales [16]. The higher the coefficient (ranging from 0 to 1), the more consistent the scale is, and the greater the likelihood that it measures a single underlying variable in the questionnaire. A value of ≥0.7 indicates high reliability; 0.5 to <0.7, moderate reliability; >0.2 to <0.5, low reliability; and ≤0.2, very low reliability.

Principal component analysis was conducted to determine whether the SQoL questionnaire after PSD surgery is unidimensional and whether some of its questions could be removed.

Test–retest reliability was estimated using Spearman’s rank correlation coefficients (rho) from two assessments conducted 1 week apart (1 week and 2 weeks postoperatively). Correlation was considered “strong” (rho > 0.70), “moderate” (0.40 < rho < 0.69), or “weak” (rho < 0.39) [17].

Evidence for construct validity was obtained from a priori hypothesised associations with other validated instruments used to measure comparable constructs (positive correlations) [18]. The validity of the SQoL questionnaire after PSD surgery was determined by comparing its results with different SF-36 domain scores using Spearman’s rank correlation coefficient (rho), consistent with previously reported correlations.

Discriminant validity was assessed by comparing the results of different domains of the SQoL questionnaire after PSD surgery for patients who were either satisfied or dissatisfied two months after surgery. A dissatisfied result was considered when patients were diagnosed with a non-healing postoperative wound after two months. Mean values were compared using the Mann–Whitney U test.

## 3. Results

### 3.1. Demographic Characteristics

In total, 116 patients who underwent surgery for PSD were included in the study. They were asked to fill out the SQoL and SF-36v2 questionnaires. Basic characteristics of the participants are shown in Table 1. They were surveyed before surgery, at first and second weeks, and 2 months after surgery. After 2 months, 103 surveys were collected (88.79% response rate). Detailed questionnaire response rates and acceptability results are presented in Table 2.

### 3.2. Reliability

Internal consistency was calculated 1 week after surgery, with the overall Cronbach’s α coefficient being 0.919. After removing any single variable, the Cronbach’s α coefficient ranged from 0.912 to 0.919. The coefficients are presented in Table 3.

Internal consistency was evaluated in each of the 4 subscales. The Cronbach’s α coefficient ranged from 0.764 in the wound subscale to 0.931 in the pain subscale. The data are presented in Table 4.

### 3.3. Principal Component Analysis

Prior to exploratory factor analysis, the suitability of the data for factor analysis was assessed. The Kaiser–Meyer–Olkin (KMO) measure of sampling adequacy was 0.872, indicating very good adequacy of the correlation matrix for factor analysis. Bartlett’s test of sphericity was statistically significant (*p* < 0.001), confirming that the correlations among items were sufficient to justify factor extraction.

Four components with eigenvalues greater than 1 were identified, explaining 68.18% of the total variance. The first component was the most important, accounting for 44.45% of the total variance (Table 5). All factor loadings for the first component were >0.4 (except for item Nr.18) in the unrotated solution (Table 6). Factor rotation was subsequently performed using the Varimax method with Kaiser Normalisation (Table 7).

### 3.4. Test–Retest Reliability

In total, 108 participants (93.1%) completed the questionnaire after 2 weeks. The Spearman correlation coefficient showed a moderate correlation of 0.55 (CI 0.406–0.679) for the total questionnaire score in the test–retest validation. The correlation coefficients for individual questions between the one and two-week post-surgery administrations ranged from 0.530 to 0.776. This correlation was significant for all questions. This correlation is indicated in Table 8.

Questionnaire reliability was assessed using the split-half method. The Spearman–Brown (0.732) and Guttman split-half (0.730) coefficients demonstrated moderate but acceptable reliability, indicating a satisfactory level of agreement between the two halves of the questionnaire.

### 3.5. Construct Validity

Construct validity was determined by comparing the SQoL with the results of different domains of SF-36v2 1 week after the surgery. Spearman’s correlation coefficient shows the inverse correlation between different domains of SF-36v2 and the overall result of the SQoL, ranging from −0.264 to −0.770. The highest correlations were observed between the corresponding domains Physical functioning and Role physical functioning (−0.770 and −0.600). All correlations were statistically significant. All correlations between different domains of SF-36v2 and the SQoL are presented in Table 9.

### 3.6. Discriminant Validity

The discriminant validity analysis revealed that after 2 months, 24 out of 103 (23.30%) patients had an unhealed wound in the sacrococcygeal area and were not satisfied with their quality of life. The mean values of all subscales and the overall score of the SQoL were significantly (*p* < 0.001) lower for satisfied patients compared to dissatisfied patients. These values are shown in Table 10. Significant differences between satisfied and dissatisfied patients were also found in many SF-36v2 domains, except for General Health, Vitality, Role Emotional, Mental Health, and the Mental Component Summary Score (Table 11).

### 3.7. Unsuccessful Surgical Outcomes

Due to the short follow-up period, new sinus openings, wound discharge after complete healing, chronic wounds after excision, and any kind of repeat surgery were classified as surgical failures rather than recurrences [2].

Six months after surgery, 90 patients returned for a follow-up examination, with unsuccessful surgical outcomes observed in 9 patients (10%). A comparison of QoL outcomes between healthy patients and those with unhealed wounds revealed statistically significant differences in pain, wound subscales, and overall QoL. Quality of Life was also assessed separately based on the surgical method used; however, due to the small sample size and the low number of failed surgeries in individual subgroups, no statistically significant difference was found. Table 12 presents the median QoL scores for both groups at the six-month mark.

## 4. Discussion

Evaluating the quality of life (QoL) after treatment for PSD presents several challenges that underscore the need for a specialised assessment tool tailored to this condition. Pilonidal sinus disease, characterised by recurrent abscesses or sinus tracts in the sacrococcygeal area, primarily affects adolescents and young adults with a peak incidence between 13 and 22 years [3]. The symptoms, which often include pain, wound care management, and potential complications like failure of treatment or infection, can significantly impact daily activities, physical comfort, negatively affect sexual function and emotional well-being, thereby diminishing quality of life [4,5].

The impact of the disease cannot be comprehensively addressed by generic QoL instruments designed for general health assessments. Studies often assess the impact of the disease and its treatment on QoL using visual analogue scale (VAS) and SF-36v2 scores. Pronks et al.’s study comparing short-term outcomes of different treatment methods utilised both scales to evaluate treatments. Variations in the results indicate that standard QoL questionnaires do not sufficiently capture the disease-specific symptoms and their impact on patients’ quality of life after treatment [14]. Similarly, a recent study by Cassaro et al. demonstrated that generic assessment tools encounter similar limitations when assessing the distinct surgical and postoperative recovery profiles of adolescents and children undergoing treatment for PSD [15].

A disease-specific QoL instrument tailored for PSD incorporates items that specifically address symptoms unique to the condition. The findings of this study demonstrate that the adapted questionnaire is both reliable and valid, with multiple lines of evidence supporting its use in clinical practice. Notably, the instrument exhibited excellent internal consistency, as reflected by high Cronbach’s α coefficients. Principal component analysis and test–retest analysis further supported the questionnaire’s moderate reliability, demonstrating that each item was appropriate and contributed meaningfully to the overall assessment. However, the interpretation of the test–retest results should be made with caution, as the assessment interval coincided with a period during which meaningful clinical improvement is expected, meaning that observed changes may reflect both measurement stability and true postoperative recovery. In addition, the Spearman–Brown and Guttman split-half coefficients indicated moderate but acceptable reliability, demonstrating satisfactory agreement between the two halves of the questionnaire.

Construct validity is supported by strong correlations with relevant SF-36v2 subscales, indicating that the questionnaire appropriately captures domains related to the quality of life of patients with PSD. Discriminant validity analyses further demonstrated statistically significant differences between patients satisfied with their quality of life and those who were not, highlighting the instrument’s ability to distinguish between different levels of perceived health status. Together, these findings provide robust psychometric evidence supporting the reliability and validity of the adapted questionnaire and reinforce its usefulness in clinical settings [19].

Furthermore, the questionnaire may help to identify early unsuccessful surgical outcomes, offering a more nuanced evaluation of treatment effectiveness. However, due to the limited number of unsuccessful cases and the lack of formal predictive analyses, the current findings indicate an association rather than true predictive capability. This may help clinicians and researchers better understand challenges during recovery and support the development of more personalised care strategies aimed at improving postoperative patient satisfaction and well-being.

Another issue is the lack of a standardised classification system [12]. Lacking such a system, variations in the selection of the treatment are prone to unacceptable levels of selection bias, thus, comparison of outcomes in these studies is not logical [20,21,22]. Implementing standardised outcome measures could improve the reliability of meta-analyses and systematic reviews.

The study possesses certain inherent limitations, including potential memory and response biases. First, the response rate was 88.79%, although the patients were repeatedly contacted by email and phone to participate. Second, the inability to resume work might have been impacted by cultural factors and the standardised duration of medical leave prescribed by family physicians. Third, including only adults excludes a substantial portion of patients who develop symptomatic PSD before the age of 18. As discussed previously, generic assessment tools fail to capture disease-specific symptoms and their impact on quality of life following treatment in paediatric and adolescent patients. While disease-specific questionnaires may address some of these limitations, the questionnaire in question was developed and validated exclusively for adults. As a result, its metrics may not adequately reflect the experiences and outcomes of children and adolescents. Therefore, further modifications are required to ensure that the instrument is appropriate, relevant, and valid for use in this younger population.

Although significantly fewer women than men (18.1% and 81.9% respectively) participated in the study, this aligns with the natural gender prevalence of the disease [23]. Therefore, this disparity was not considered a limitation.

Additionally, the study’s setting itself may have influenced the outcomes. It should be noted that this was a single-centre study primarily consisting of adult participants from a homogeneous ethnic group. No cross-cultural adaptation, external validation cohort, or multicentre assessment was performed. Moreover, the patients received procedures that are approved and most commonly performed by surgeons at Kaunas Hospital, which excludes some potentially beneficial procedures that have not yet been adopted by the hospital’s surgeons. Moreover, the follow-up period was 6 months. To obtain more robust and generalisable results, further randomised studies with larger sample sizes and extended follow-up periods are required. By addressing these limitations, we can gain a better understanding of the effectiveness and impact of various treatment strategies on patient outcomes.

## 5. Conclusions

A newly developed SQoL questionnaire has shown promise in assessing the impact of the disease and its treatment, particularly by addressing specific symptoms. Standardising outcome measures can improve the reliability of meta-analyses and systematic reviews, which in turn helps to create more personalised care plans.

## Figures and Tables

**Table 1:** Basic characteristics.

Characteristics	N-116
Age (Mean ± SD)	29.10 ± 9.26
Sex N (%)	
Male	95 (81.9%)
Female	21 (18.1%)
BMI (Mean ± SD)	27.32 ± 4.87
ASA Physical status *n* (%)	
I	105 (90.5%)
II	11 (9.5%)
Surgery type *n* (%)	
Excision	50 (43.1%)
Minimal invasive pit-picking treatment	62 (53.4%)
Excision m. Karydakis	4 (3.5%)
Pilonidal sinus disease character *n* (%)	
Primary	90 (77.6%)
Recurrent	26 (22.4%)

**Table 2:** Participant response and completion rates (%).

	SQoL	SF-36v2
Before surgery		
Received	116 (100%)	116 (100%)
Fully completed	116 (100%)	114 (98.3%)
First week after surgery		
Received	110 (94.8%)	110 (94.8%)
Fully completed	110 (94.8%)	110 (94.8%)
Second week after surgery		
Received	110 (94.8%)	110 (94.8%)
Fully completed	108 (93.1%)	108 (93.10%)
Second month after surgery		
Received	103 (88.8%)	103 (88.8%)
Fully completed	103 (88.8%)	103 (88.8%)

**Table 3:** Reliability Scale (Cronbach’s α if item was deleted).

Item	Corrected Item-Total Correlation	Cronbach’s α If Item Deleted
Nr. 1	0.651	0.914
Nr. 2	0.686	0.912
Nr. 3	0.713	0.913
Nr. 4	0.723	0.912
Nr. 5	0.699	0.912
Nr. 6	0.670	0.913
Nr. 7	0.677	0.913
Nr. 8	0.742	0.912
Nr. 9	0.725	0.913
Nr. 10	0.562	0.915
Nr. 11	0.430	0.919
Nr. 12	0.416	0.919
Nr. 13	0.509	0.917
Nr. 14	0.405	0.919
Nr. 15	0.701	0.912
Nr. 16	0.476	0.917
Nr. 17	0.531	0.916
Nr. 18	0.578	0.915
Nr. 19	0.623	0.914
Nr. 20	0.509	0.917

**Table 4:** Reliability Scale (Cronbach’s α of the 4 subscales).

Subscale	Cronbach’s α	Inter-Item Correlation (Min–Max)	Inter-Item Correlation (Mean)
Pain	0.931	0.617–0.847	0.732
Physical activity	0.898	0.497–0.875	0.664
Wound	0.764	0.269–0.718	0.394
Psychosocial	0.802	0.313–0.772	0.445

**Table 5:** Principal component analysis.

Initial Eigenvalues			Extraction Sums of Squared Loadings			Rotation Sums of Squared Loadings		
Total	Variance	%	Total	Variance	%	Total	Variance	%
8.889	44.45	44.45	8.89	44.45	44.45	6.56	32.80	32.80
2.203	11.02	55.46	2.20	11.01	55.46	3.18	15.90	48.70
1.509	7.54	63.00	1.51	7.54	63.01	2.39	11.96	60.66
1.036	5.18	68.18	1.03	5.18	68.18	1.51	7.53	68.18

**Table 6:** Component Matrix.

Item	Component 1	Component 2	Component 3	Component 4
Nr. 1	0.820	−0.328	0.199	0.108
Nr. 2	0.816	−0. 260	0.073	0.181
Nr. 3	0.813	−0.217	−0.075	−0.172
Nr. 4	0.812	−0.102	−0.041	−0.130
Nr. 5	0.807	−0.323	0.079	0.207
Nr. 6	0.790	−0.331	−0.003	−0.337
Nr. 7	0.781	−0.273	0.031	0.307
Nr. 8	0.775	−0.272	0.043	−0.360
Nr. 9	0.734	−0.174	0.164	0.150
Nr. 10	0.700	0.312	−0.100	0.145
Nr. 11	0.648	−0.189	−0.134	−0.020
Nr. 12	0.622	0.384	−0.468	−0.058
Nr. 13	0.587	0.339	−0.546	−0.006
Nr. 14	0.560	0.213	−0.045	−0.190
Nr. 15	0.516	0.352	−0.462	0.009
Nr. 16	0.466	0.491	0.072	−0.073
Nr. 17	0.424	0.467	0.119	0.238
Nr. 18	0.391	0.502	0.544	−0.238
Nr. 19	0.503	0.461	0.543	−0.236
Nr. 20	0.426	0.296	0.221	0.555

**Table 7:** Rotated Component Matrix.

Item	Component 1	Component 2	Component 3	Component 4
Nr. 1	0.868	0.049	0.150	0.232
Nr. 2	0.809	0.126	0.033	0.294
Nr. 3	0.783	0.324	0.150	−0.056
Nr. 4	0.717	0.354	0.221	0.019
Nr. 5	0.837	0.126	0.033	0.294
Nr. 6	0.845	0.214	0.187	−0.226
Nr. 7	0.778	0.167	−0.016	0.382
Nr. 8	0.805	0.206	0.256	−0.225
Nr. 9	0.704	0.111	0.174	0.285
Nr. 10	0.369	0.535	0.279	0.344
Nr. 11	0.621	0.295	0.016	0.041
Nr. 12	0.236	0.821	0.143	0.079
Nr. 13	0.223	0.836	0.036	0.089
Nr. 14	0.334	0.407	0.346	−0.006
Nr. 15	0.166	0.748	0.068	0.110
Nr. 16	0.103	0.411	0.506	0.180
Nr. 17	0.073	0.323	0.385	0.459
Nr. 18	0.091	0.050	0.856	0.122
Nr. 19	0.206	0.081	0.865	0.137
Nr. 20	0.169	0.139	0.225	0.726

A variable is associated with a factor if its factor > 0.4.

**Table 8:** Test–retest reliability.

Question	Spearman’s Rho	*p* Value	95% Confidence Intervals	
			Lower	Upper
Nr. 1	0.557	<0.001	0.406	0.679
Nr. 2	0.716	<0.001	0.605	0.799
Nr. 3	0.563	<0.001	0.413	0.683
Nr. 4	0.580	<0.001	0.434	0.697
Nr. 5	0.611	<0.001	0.472	0.721
Nr. 6	0.738	<0.001	0.634	0.816
Nr. 7	0.696	<0.001	0.579	0.784
Nr. 8	0.679	<0.001	0.558	0.772
Nr. 9	0.703	<0.001	0.589	0.790
Nr. 10	0.740	<0.001	0.637	0.817
Nr. 11	0.530	<0.001	0.373	0.657
Nr. 12	0.725	<0.001	0.617	0.806
Nr. 13	0.758	<0.001	0.661	0.830
Nr. 14	0.776	<0.001	0.684	0.843
Nr. 15	0.645	<0.001	0.514	0.746
Nr. 16	0.769	<0.001	0.675	0.839
Nr. 17	0.680	<0.001	0.559	0.773
Nr. 18	0.667	<0.001	0.542	0.763
Nr. 19	0.636	<0.001	0.503	0.740
Nr. 20	0.666	<0.001	0.541	0.762

**Table 9:** Correlation between the SQoL questionnaire and SF-36v2.

Category	Correlation	*p* Value
Physical functioning	−0.770	<0.001
Role physical functioning	−0.600	<0.001
Bodily pain	−0.507	<0.001
General Health	−0.320	0.001
Vitality	−0.294	0.002
Social functioning	−0.592	<0.001
Role emotional	−0.371	<0.001
Mental Health	−0.264	0.005

**Table 10:** SQoL scores (mean ± SD) for satisfied and dissatisfied participants.

Subscale	Satisfied	Dissatisfied	*p* Value
Pain	0.52 ± 1.42	2.52 ± 3.87	<0.001
Physical activity	1.13 ± 3.77	2.61 ± 3.02	<0.001
Wound	2.54 ± 4.68	9.87 ± 6.01	<0.001
Psychosocial	1.76 ± 3.53	5.65 ± 5.04	<0.001
Total	5.95 ± 9.97	20.65 ± 12.81	<0.001

**Table 11:** SF-36v2 scores (mean ± SD) for satisfied and dissatisfied participants.

Category	Satisfied	Dissatisfied	*p* Value
Physical functioning	94.21 ± 12.06	81.30 ± 19.95	<0.001
Role physical functioning	87.30 ± 32.02	68.47 ± 38.59	0.004
Bodily pain	91.35 ± 14.03	84.05 ± 16.69	0.021
General Health	69.60 ± 20.18	63.47 ± 20.85	0.219
Vitality	73.41 ± 14.69	67.17 ± 18.07	0.101
Social functioning	92.23 ± 14.75	84.05 ± 19.77	0.047
Role emotional	87.83 ± 28.89	72.46 ± 42.22	0.070
Mental Health	76.12 ± 15.99	76.69 ± 14.30	0.984
Physical component summary score	85.59 ± 13.90	74.32 ± 16.62	0.001
Mental component summary score	82.40 ± 14.41	75.09 ± 18.33	0.097

**Table 12:** QoL 6 months after surgery for patients with and without unsuccessful surgical outcomes.

Subscale	Successful Outcomes N-81	Unsuccessful Outcomes N-9	*p* Value
Pain	0 (−0.04–0.32)	1 (−1.21–5.50)	<0.001
Physical activity	0 (−0.01–1.48)	0 (−0.83–1.97)	0.787
Wound	0 (1.24–3.14)	3 (0.03–14.82)	0.005
Psychosocial	0 (0.80–2.29)	1 (−0.92–8.92)	0.160
Total	0 (2.62–6.59)	7 (−1.75–30.07)	0.025

Median (95% Confidence Interval).

## Data Availability

The data presented in this study are available on request from the corresponding author.

## Supplementary Materials

The following supporting information can be downloaded at: https://www.mdpi.com/article/10.3390/jcm15155875/s1, Supplementary Table S1: Pilonidal Disease Quality of Life Questionnaire.

## Abbreviations

The following abbreviations are used in this manuscript:

LUHS	Lithuanian University of Health Sciences
PSD	Pilonidal sinus disease
QoL	Quality of life
SQoL	Disease-specific quality of life
VAS	visual analogue scale
ASA	American Society of Anesthesiologists
