# Supplementary material for: The Pilonidal Sinus Disease-Specific Quality of Life Questionnaire (SQoL): Reliability, Validity, and Utilisation
Source: J Clin Med. 2026 Jul 27;15(15):5875. doi: 10.3390/jcm15155875 (PMC13466662; doi:10.3390/jcm15155875)
Supplement: Supplementary file 1 [file jcm-15-05875-s001.zip › jcm-4341243-supplementary.pdf]

Supplementary Table S1 Pilonidal Disease Quality of Life Questionnaire

I. The following questions relate to pain associated with pilonidal disease. Please indicate the intensity of pain you experience.

Statements	Not at all	Very mild	Mild	Moderate	Severe	Very severe
I experience pain at rest.						
I experience pain while sitting (including on the toilet).						
I experience pain while walking (a few hundred meters).						
I experience pain during light daily activities (getting out of bed, washing, dressing).						
I experience pain during moderately demanding activities (vacuuming).						

II. The following questions relate to your physical activity. Please indicate how your current condition (pilonidal disease) has affected these activities.

Statements	No change	Slightly changed	Moderately changed	Considerably changed	Severly changed
Walking (a few hundred meters).					
Climbing stairs (several flights).					
Light daily activities (dressing, washing, bathing, household chores).					
Moderately demanding activities (vacuuming, gardening).					
Vigorous activities (running, lifting heavy objects).					

III. The following questions relate to discomfort associated with pilonidal disease or postoperative wound care. Please indicate the extent to which you agree with the statements.

Statements	False	Mostly false	Neither true nor false	Mostly true	True
Dressing changes are painful.					
I feel dependent on others for wound care.					
Wound discharge (leakage) causes me discomfort.					
The wound causes an unpleasant odor.					
The condition restricts my movements.					

IV. The following questions relate to your emotional well-being, social functioning, and sexual function. Please indicate your level of agreement with the statements.

**Statements**	**False**	**Mostly false**	**Neither true nor false**	**Mostly true**	**True**
I constantly worry about the treatment of my condition.					
Due to this condition, I spend less time on work or other activities.					
Due to this condition, I spend less time on social activities and leisure (attending public events, meeting friends).					
This condition has reduced my sexual activity.					
Due to this condition, I experience discomfort during sexual intercourse.					
